# Exaggerated IFN-I Response in Long COVID PBMCs Following Exposure to Viral Mimics

**DOI:** 10.1007/s10875-025-01969-w

**Published:** 2025-12-10

**Authors:** Bart Humer, Julia C. Berentschot, Cornelia G. van Helden-Meeuwsen, L. Martine Bek, Maaike de Bie, Tobias M. Defesche, Chantal A. Boly, Manon Drost, Merel E. Hellemons, Willem A. Dik, Marjan A. Versnel

**Affiliations:** 1 Department of Immunology, Erasmus MC, University Medical Center Rotterdam, P.O Box 2040, 3000 CA, Rotterdam, The Netherlands; 2 Department of Respiratory Medicine, Erasmus MC, University Medical Center Rotterdam, Rotterdam, The Netherlands; 3 Reinier Hage Medisch Diagnostisch Centrum (RHMDC), Laboratory Medical Immunology, Delft, The Netherlands; 4 Department of Intensive Care, Erasmus MC, University Medical Center, Rotterdam, The Netherlands

**Keywords:** Long COVID, PASC, Fatigue, Type I interferon, Innate immune memory, Epigenetic memory, cGAS, RIG-I

## Abstract

**Purpose:**

Long COVID (LC) is a long-term debilitating disease of which the exact pathophysiology is unknown. A dysregulated immune response resulting in hyperresponsive immune cells is hypothesized as a key mechanism in the development of LC. Several studies suggest that acute infections can leave lasting epigenetic changes, which result in heightened immune reactivity. Upon stimulation, these primed immune cells may exhibit exaggerated responses. This form of epigenetic memory can contribute to altered immune dynamics, particularly in response to induction of type I Interferons (IFN-I) pathway activation using a viral mimic. Therefore, we investigated if LC patients exhibit a hyperresponsive response towards viral mimics in comparison with healthy controls (HC).

**Methods:**

PBMCs of two distinct LC cohorts, characterized by a different disease course and duration, were collected and transfected using Lyovec with the cGAS and RIG-I agonists G3-YSD and 3p-RNA followed by measurement of IFN-I bioactivity with a reporter cell line.

**Results:**

Transfection of PBMCs of LC patients with the cGAS and RIG-I agonists resulted in increased IFN-I bioactivity in comparison with HC. Unsupervised hierarchical clustering revealed two distinct clusters, each predominantly composed of either patients or HC. In addition, a moderate correlation between RIG-I stimulation with 3p-RNA and fatigue severity scores was found.

**Conclusion:**

These data show a hyperresponsive phenotype of immune cells of LC patients upon stimulation with viral mimics. The current availability of biologicals and small molecule inhibitors that interfere with aberrant IFN-I pathway activation underscores the importance of pursuing future investigations into this phenomenon.

**Graphical Abstract:**

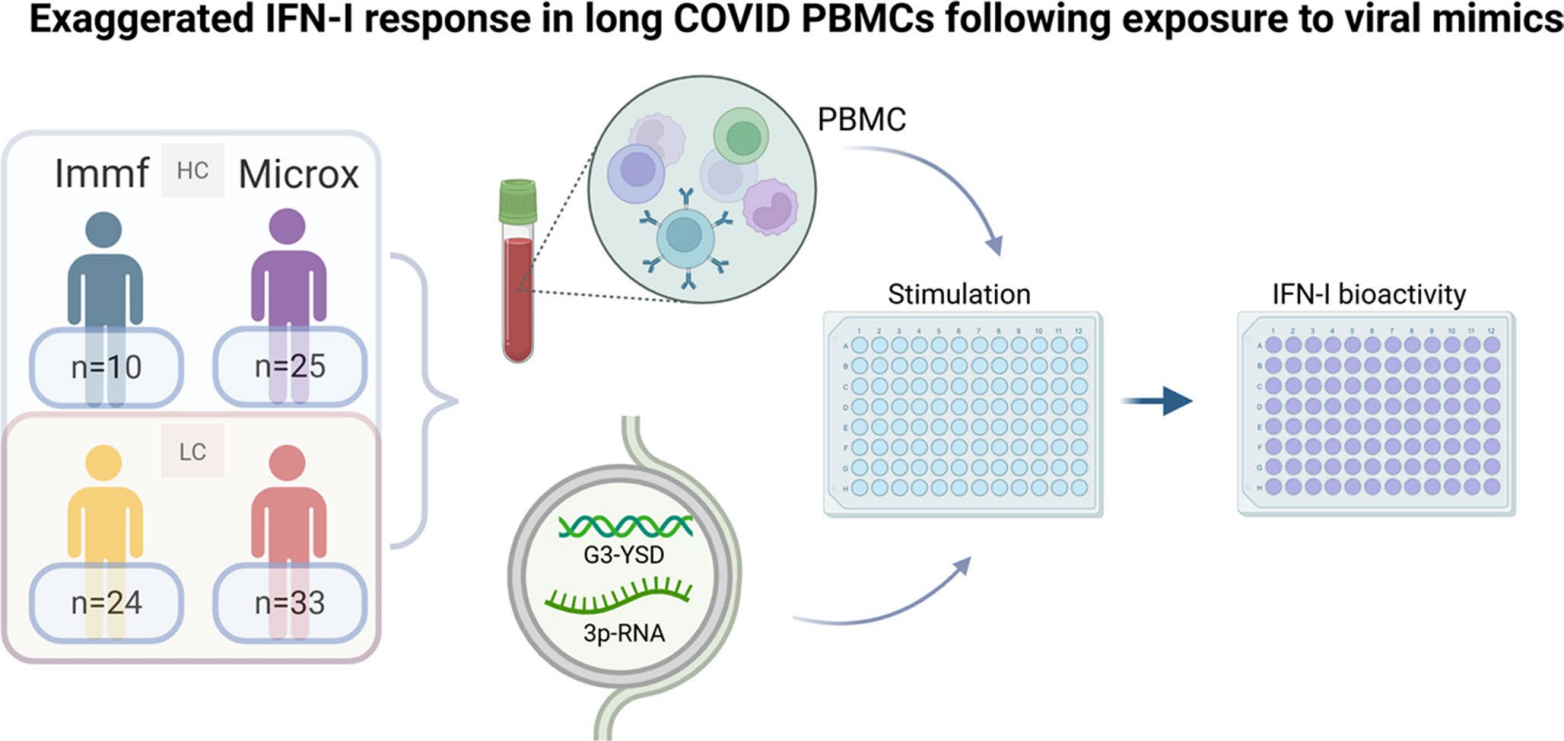

**Supplementary Information:**

The online version contains supplementary material available at 10.1007/s10875-025-01969-w.

## Introduction

Long COVID (LC) or post-acute sequelae of SARS-CoV-2 (PASC) presents with a wide range of clinical symptoms, such as fatigue, post-exertional malaise (PEM), and cognitive impairments, that can persist for months or even years after SARS-CoV-2 infection [1, 2]. The exact mechanisms underlying LC are poorly understood. However, a dysregulated immune response is commonly cited as a key mechanism in developing LC [3]. This immune imbalance may involve hyperactive immune cell responses upon activation, leading to an excessive production of inflammatory mediators, which could contribute to LC [4].

Studies have shown that innate immune cells and their progenitors can retain an epigenetic memory of prior infections or inflammatory conditions [5–7]. This epigenetic memory is preserved in hematopoietic precursor cells, causing their progeny to inherit these epigenetic changes and ultimately resulting in a persistent hyperresponsive immune profile [5–7]. Cheong et al., found that convalescent COVID-19 patients exhibit alterations in epigenetic and transcriptional programs of hematopoietic stem cells and their descendant monocytes [8]. These alterations were linked to increased myelopoiesis and the development of more inflammatory monocytes [8], potentially enhancing production of type I Interferon (IFN-I), which can further reinforce and propagate epigenetic reprogramming in immune cells.

IFN-I are critical immunomodulatory cytokines, required to combat SARS-CoV-2. Interestingly, the immunomodulatory effect of IFN-I can be retained by histone modifications serving as transcriptional memory in PBMCs [9–11]. This implies that subsequent stimulation may invoke higher IFN-I induction. Whether this relates to LC symptoms remains unknown.

Considering the long-lasting effects of acute infections on IFN-I inducibility [9–11], we hypothesized that LC patients exhibit a hyperresponsive anti-viral immune response upon stimulation compared to non-LC controls. To investigate this, we mimicked viral activation in PBMCs by triggering the cytosolic RNA- and DNA sensing receptors RIG-I and cGAS and subsequently measured IFN-I bioactivity.

## Methods

### Participants

PBMC were collected from two cohort studies, both containing adult LC and control groups, conducted in Erasmus MC Rotterdam.

#### Immunofatigue Cohort

LC patients previously hospitalized with confirmed COVID-19 (discharged between October 2020 and May 2021). PBMC were collected during outpatient clinic visits 3–6 months post-discharge. Age- and sex-matched healthy controls without substantial fatigue and depressive symptoms were recruited. This cohort has been described in detail previously [12]. For the current study, PBMCs from 24 LC patients (Immf-LC) and 10 controls (Immf-HC) were analyzed.

#### MICROX Cohort

LC patients and age- and sex-matched convalescent controls were recruited between December 1, 2023 and February 21, 2024. LC patients were previously healthy individuals, had a confirmed prior COVID-19 diagnosis, had a LC diagnosis > 6 months, and experienced PEM. Mean LC duration was 3.2 [2.0–3.8] years. Convalescent controls had confirmed prior COVID-19 diagnosis but had an uneventful recovery without persistent symptoms. All participants were surveyed for vaccination status and reinfection, which were required to have occurred at least three months before inclusion. For the current study, PBMCs from 33 LC patients (Microx-LC) and 25 controls (Microx-HC) were analyzed.

Both cohorts performed extensive clinical characterization of the participants using a range of patient-reported outcome measures (PROMs), and required participants to be sufficient in either the Dutch or English language. All participants provided written informed consent before the start of the study measurements. The Medical Ethics Committee of Erasmus MC approved the studies (Imunofatigue MEC-2020-1893; Microx: MEC-2023-0588).

### Blood Sampling, Processing and PBMC Stimulation

Peripheral blood was obtained in heparine/EDTA followed by PBMCs isolation and cryopreservation. PBMCs were thawed and plated at a density of 3.0 × 10^5^ in a volume of 50 ul in 10% FBS RPMI Medium 1640 with glutamax, penicillin and streptomycin (Gibco, Thermo Fisher Scientific) into 96-well round bottom plates (Nunclon Della plates; Thermo Fisher Scientific). To investigate IFN-I production by PBMCs, IFN-I bioactivity was measured before and after mimicking a viral infection using LyoVec (Invivogen) with G3-YSD (cGAS agonist, 1 mg/ml, Invivogen), or LyoVec with 3pRNA (RIG-I agonist, 1 mg/ml, Invivogen), followed by an incubation period of 24 h in a humidified incubator at 37 C/5% CO_2_. Supernatant was harvested and stored at −20 °C until IFN-I bioactivity analysis.

### Composition of PBMC Fractions

The cellular composition of the used PBMC fractions in our study was obtained from flowcytometric analysis, more specifically the proportion of monocytes (classical, intermediate and non-classical), total T-cells, CD4 + T-cells, CD8 + T-cells, total B-cells and NK-cells, as described previously [13].

### Type I IFN Bioactivity Analysis

IFN-I bioactivity was determined with a reporter assay system (HEK-Blue™ IFN-α/β cells; InvivoGen) as previously described [14].

### Statistical Analysis

Study samples across cohorts were chosen by random sampling through RStudio.

A Kruskal-Wallis test was used to compare groups, with an uncorrected post-hoc Dun’s test. A Spearman correlation test was used to assess correlations between parameters. Statistical significance was indicated by *p* < 0.05. A heatmap of logarithmically scaled IFN-I bioactivity was generated using the ‘pheatmap’ package in RStudio (R 4.4.2) for Lyovec, 3p-RNA, and G3-YSD stimulation. Hierarchical clustering was performed using Euclidean distance and the ward.D2 method, with dendrograms illustrating clustering relationships.

## Results

**Fig. 1:**
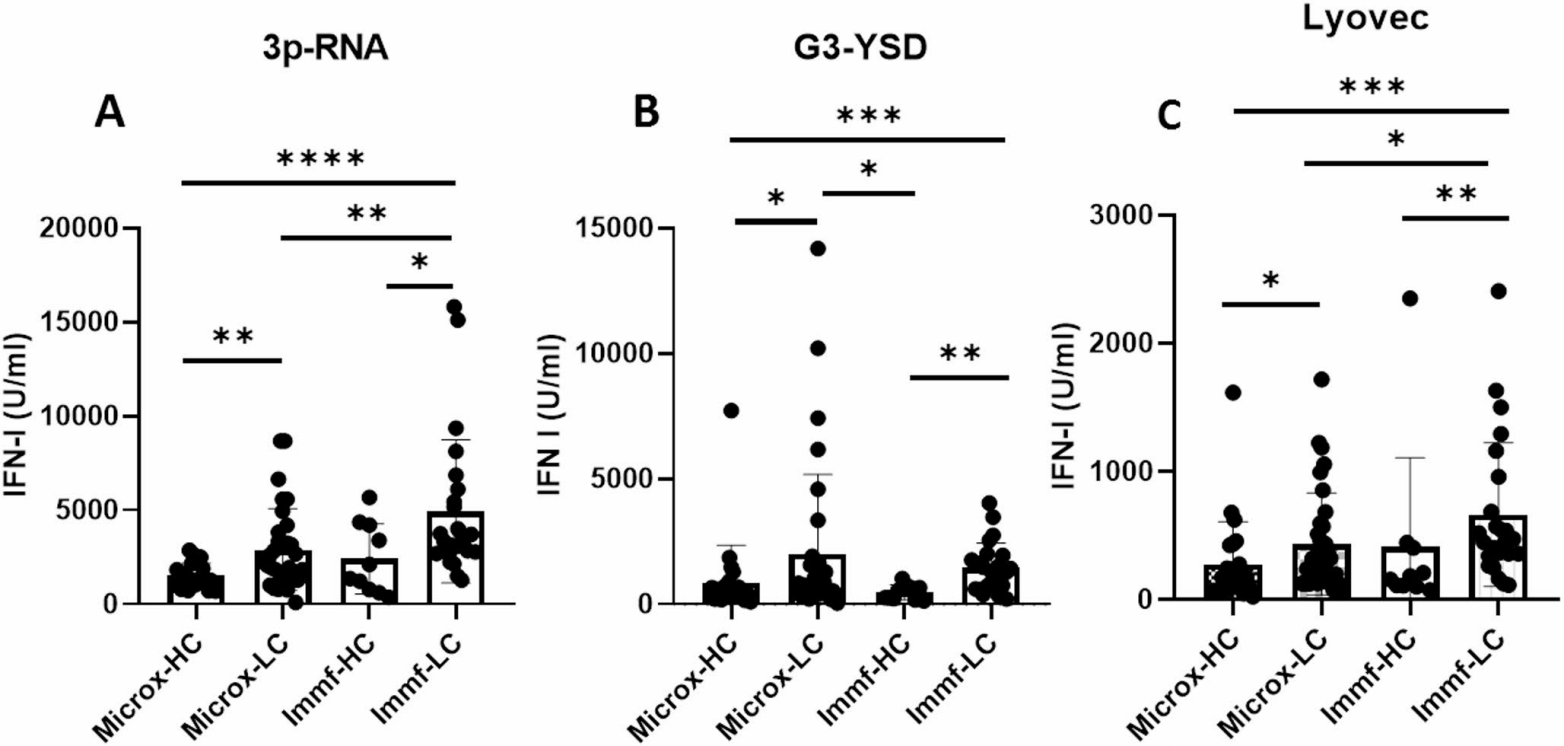
IFN-I bioactivity measured by a reporter assay after 24 h of stimulation with Lyovec in combination with 3p-RNA (**A**), Lyovec in combination with G3-YSD (**B**), and Lyovec in isolation (**C**). Bars represent the mean and error bars indicate standard deviations. *p* < 0.01 (*), *p* < 0.01 (**), *p* < 0.001 (***), *p* < 0.0001 (****)

To augment the generalizability of our findings, we included two distinct LC cohorts differentiated by disease course and duration (Table 1). Stimulation with the RIG-I agonist 3p-RNA revealed significant differences in IFN-I bioactivity between Microx-HC and Microx-LC, as well as immf-LC and both HC groups (Fig. 1A), while no significant differences were observed among HCs. A similar pattern was observed for cGAS activated IFN-I bioactivity, where Microx-LC showed significantly higher IFN-I bioactivity compared to both HC groups (Fig. 1B). Under basal conditions, without any stimulation, no significant differences in bioactive IFN-I production were observed between LC patients and HC within cohorts (Supplemental Fig. 1). However, exposure of PBMCs to Lyovec alone led to a significant increase in bioactive IFN-I production in Microx-LC compared to Microx-HC, as well as Immf-LC compared to Immf-HC (Fig. 1C). Between cohorts, Immf-LC also showed more exaggerated responses compared to Microx-HC (Fig. 1C). Supplementary Fig. 2 illustrates the percentage of patients exhibiting increased IFN-I responses. Given the role of monocytes in the type I IFN response, we measured total monocytes and their subsets in the PBMC population. No significant differences were observed within the Microx and Immunofatigue cohort (Supplementary Figs. 3 & 4). Additional PBMC analysis are shown in Supplementary Fig. 5. Participant age did not significantly influence the results (data not shown).

**Table 1:** Clinical characteristics of included participants

		Immunofatigue				Microx		
	*n*^a^	Long COVID **(n** = 24)	n	**Controls (n = 10)**	*n*	Long COVID **(n = 33)**	*n*	Controls **(n** = 25)
**Patient characteristics**								
Age, years (mean + SD and median + IQR)		58.5 ± 12.2 62.0 [54.0–66.0]		55.3 ± 16.0 61.0 [39.0–68.0]		41.9 ± 10.4 38.0 [34.0-51.5]		40.9 ± 10.2 39.0 [31.5–49.0]
Sex, male		13 (54%)		5 (50%)		17 (52%)		14 (56%)
BMI, kg/m² (mean + SD and median + IQR)	22	30.8 ± 5.4 31.6 [26.1–34.3]			32	24.0 ± 3.9 24.5 [20.8–26.0]		25.1 ± 3.2 24.8 [22.6–26.8]
Ethnicity					32			
European		18 (75%)				30 (94%)		24 (96%)
Non-European		6 (25%)				2 (6%)		1 (4%)
Education					32			
Low		7 (29%)				0		2 (8%)
Middle		10 (42%)				5 (16%)		7 (28%)
High		7 (29%)				27 (84%)		16 (64%)
Pre-COVID-19 Employed		14 (58%)			32	30 (94%)		23 (92%)
Medical history					32			
Obesity (BMI ≥ 30 kg/m^2^)	22	13 (59%)				3 (9%)		1 (4%)
Diabetes		6 (25%)				0		0
Cardiovascular disease or hypertension		12 (50%)				0		0
Pulmonary disease		8 (33%)				10 (31%)		3 (12%)
Autoimmune disease		0				4 (13%)		0
Mental disorder		0				12 (38%)		1 (4%)
**COVID-19 characteristics**								
Hospitalized for acute COVID-19		24 (100%)		0		0		0
ICU admission		13 (54%)						
Duration of hospital stay, days (mean + SD and median + IQR)		18.92 ± 12.6 15.5 [8.3–25.3]						
Follow-up								
Days between hospital discharge and follow-up (mean + SD and median + IQR)		126.3 ± 38.51 114.5 [98.3-162.3]						
Duration of long COVID (years)						3.1 ± 1.7 3.2 [2.0-3.8]		0
Vaccinated	22	16 (73%)		10 (100%)	32	32 (100%)		23 (92%)
**Health outcomes**								
*Symptoms*					32			
Fatigue		9 (38%)				31 (97%)		3 (12%)
Impaired fitness		19 (79%)				31 (97%)		1 (4%)
Dyspnea^b^	22	17 (77%)				21 (66%)		0
Muscle weakness		13 (54%)				20 (63%)		0
Memory problems		12 (50%)				26 (81%)		2 (8%)
Concentration problems		11 (46%)				31 (97%)		2 (8%)
Balance problems/dizziness		13 (54%)				23 (72%)		0
Sleep disturbances		11 (46%)				27 (84%)		2 (8%)
Tingling and/or pain in extremities		9 (38%)				22 (69%)		1 (4%)
Joint pain		9 (38%)				14 (44%)		1 (4%)
*Fatigue*					32			
FAS total score (mean + SD and median + IQR)		25.3 ± 9.5 23.0 [17.3–32.8]		14.9 ± 3.7 15 [11.0–18.0]		37.8 ± 7.9 39.0 [35.0-41.8]		17.8 ± 4.4 18.0 [15.0–20.0]
Fatigue (FAS ≥ 22)		12 (50%)		-		30 (94%)		4 (16%)
*Cognitive failures*					32			
CFQ total score (mean + SD and median + IQR)		30.6 ± 15.7 28 [18.8–45.5]				47.6 ± 17.4 47.5 [36.0-58.8]		20.0 ± 15.1 21.0 [6.0-28.5]
Cognitive failures (CFQ > 43)		7 (29%)				17 (53%)		2 (8%)
*Health-related quality of life*								
EQ-5D-5 L, index value (mean + SD and median + IQR)	22	0.75 ± 0.25 0.86 [0.59–0.92]			32	0.39 ± 0.22 0.44 [0.24–0.57]		0.96 ± 0.07 1.00 [0.91-1.00]

**Fig. 2:**
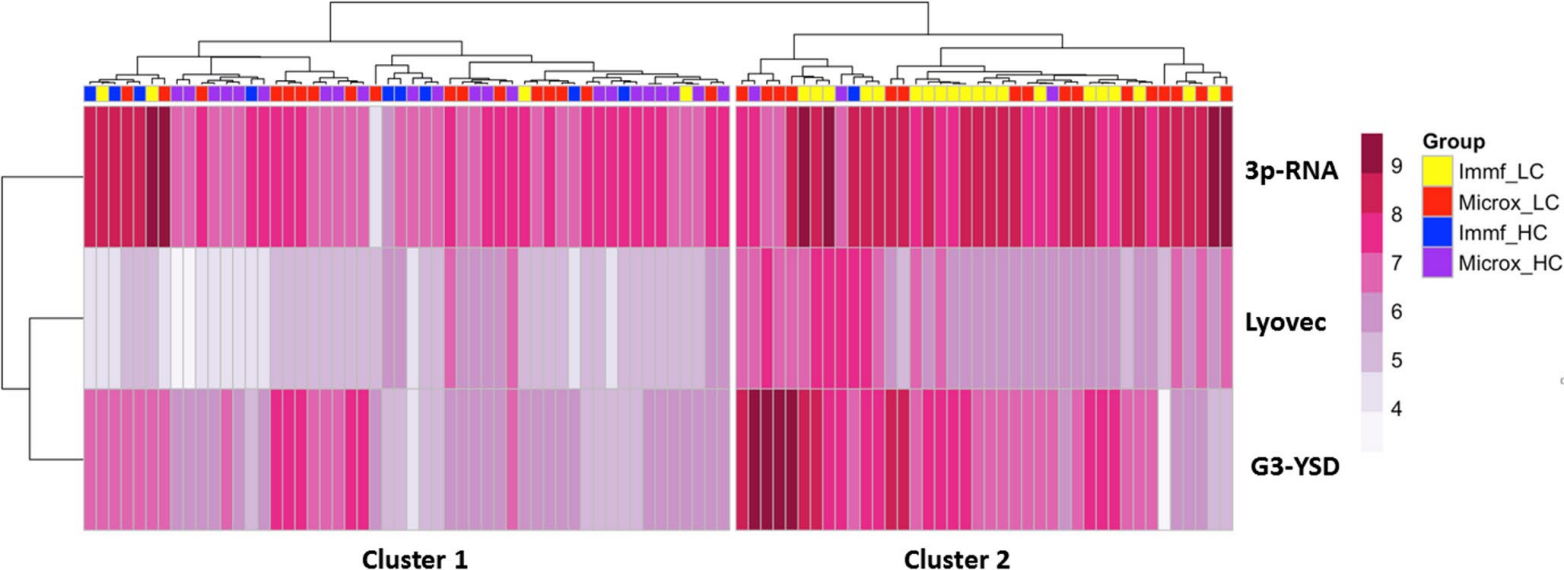
Unsupervised hierarchical clustering of patient groups based on logarithmic transformed IFN-I bioactivity after transfection using Lyovec without and with 3p-RNA, and G3-YSD. Two main clusters of IFN-I bioactivity are identified. Cluster 1 primarily comprised low HC responders for all stimulations, cluster 2 contains mostly high responders towards 3p-RNA and G3-YSD

To assess whether IFN-I bioactivity profiles were distinct between LC and HC, we performed unsupervised hierarchical clustering (Fig. 2). Cluster 1 predominantly comprised low responders to stimuli, consisting of mainly HCs 31/51 (61%). In contrast, cluster 2 consisted of mainly high responders, of whom the majority were LC patients 37/41 (90%), suggesting divergent IFN-I bioactivity profiles among LC and HC in these two cohorts. No correlation between 3p-RNA and G3-YSD IFN-I bioactivity was present (Supplemental Fig. 6).

**Fig. 3:**
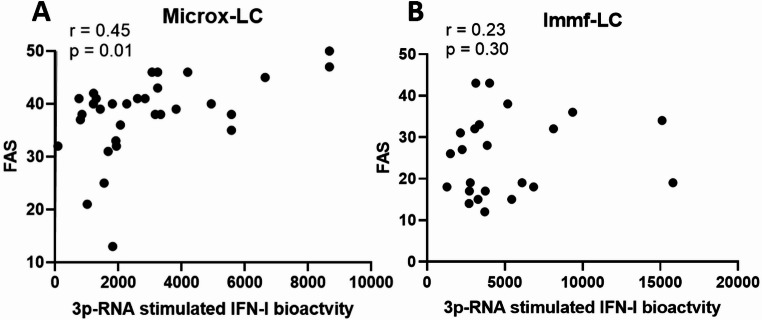
Correlation between 3p-RNA induced IFN-I bioactivity and Fatigue Assessment Scale (FAS) score for patients with long COVID (LC) in the Microx cohort (**A**) and the Immunofatigue (Immf) cohort (**B**)

Investigation of a possible relationship with fatigue severity revealed that IFN-I bioactivity (3p-RNA induced) was significantly but moderately associated with FAS scores in Microx-LC (*r* = 0.45, *p* = 0.001) (Fig. 3A). This correlation was not evident in Immf-LC (*r* = 0.23, *p* = 0.30) (Fig. 3B).

## Discussion

To our knowledge, this is the first study showing that PBMCs of LC patients exhibit exaggerated IFN-I production when exposed to viral mimics in vitro. Persistent dysregulated immune response after acute-COVID-19 is proposed as a key mechanism in the pathogenesis of LC. Upon a viral infection, such as COVID-19, IFN-I is produced when viral RNA or DNA activates the intracellular cytosolic nucleic acid sensors like RIG-I and cGAS. Alterations in one of these pathways can result in dysfunctional IFN-I transcription. A recent study in LC patients found elevated levels of cGAS-STING in peripheral blood leukocytes and an increase in IFN-I in plasma [13]. Another study found increased gene expression of the IRF family motifs IRF3, IRF5, and IRF7 in hematopoietic stem cells and descending monocytes of LC patients [8]. These data suggest increased sensitivity to activate RNA- and DNA- sensing pathways, which are in line with our observations demonstrating a hyperresponsive IFN-I axis in LC.

The persistence of SARS-CoV-2, often documented in LC [15], may have altered cellular responsiveness to IFN-I-inducing stimuli. Furthermore, SARS-CoV-2 infection has been shown to damage mitochondria, resulting in the release of mtDNA into the cytosol and subsequent activation of the cGAS-STING pathway [16, 17]. Although these hypotheses require further investigation, our findings indicate that stimulators of RIG-I and the cGAS-STING pathway may amplify IFN-I production in LC. From this observation we hypothesize that in LC a hyperresponsive type-I IFN response may occur following activation by viral particles that trigger RIG-I and/or cGAS signaling. This aligns with patient-reported clinical deterioration following infections [18].

Interestingly, therapeutic administration of IFN-I is well known to result in symptoms of fatigue supporting a role for a hyperresponsive IFN-I axis in the pathophysiology of LC [19, 20]. We found a moderate correlation between 3p-RNA IFN-I bioactivity and fatigue scores in LC, despite the inherent limitations of these PROMs, (patient reported outcome measures, such as a ceiling effect). The current availability of biologicals and small molecule inhibitors that interfere with aberrant IFN-I pathway activation might therefore be a promising treatment to explore in future studies.

The two LC groups exhibited several notable clinical differences. The Microx cohort consisted of long-term (3.2 [2.0–3.8.], years) LC patients who experienced a mild primary infection, whereas the Immunofatigue cohort included short-term (114.5 [98.3–162.3], days) LC patients who had been hospitalized during their initial illness. The overall IFN response observed was not consistently elevated in one cohort compared to the other. It remains unclear whether the disparity in responses is attributable to the more severe primary illness in the Immf-LC patients or whether prolonged illness in the Microx-LC patients led to waning of IFN responses over time. The composition of monocytes and their subsets did not differ significantly within cohorts, suggesting that the differences in response were not driven by different monocyte frequencies. Differences observed between the two cohorts, particularly in relation to the control groups, may reflect variations in cohort characteristics, such as time since vaccination and differing stages of the pandemic. Further research is necessary to clarify these differences and their implications.

In summary, our data indicate that PBMCs from LC patients exhibit an hyperresponsive IFN-I response following activation by viral mimics that trigger RIG-I and cGAS. The exact underlying mechanism that drives this requires further investigation and can guide new treatment targets.

## Supplementary Information

Below is the link to the electronic supplementary material.

Supplementary Material 1 (DOCX 339 KB)

## Data Availability

The datasets generated during and/or analyzed during the current study are available from the corresponding author on reasonable request.
