# Supplementary material for: Exaggerated IFN-I Response in Long COVID PBMCs Following Exposure to Viral Mimics
Source: J Clin Immunol. 2025 Dec 10;46(1):5. doi: 10.1007/s10875-025-01969-w (PMC12795936; doi:10.1007/s10875-025-01969-w)
Supplement: Supplementary file 1 [file 10875_2025_1969_MOESM1_ESM.docx]

Supplemental information

OLE object

Supplementary Figure 1. Total IFN-I bioactivity for both the Microx and Immunofatigue cohorts under basal condition. Bars represent the mean and error bars indicate standard deviations p < 0.01  (*), p < 0.01 (**). 

OLE object

Supplementary Figure 2. Percentage of patient with responses higher than the relative healthy control mean plus one standard deviation.  

Supplementary Figure 3. Percentage of monocytes in PBMCs of HC and LC in the Microx (A) and Immunofatigue cohort (B), Bars represent the mean and error bars indicate standard deviations, no significant differences were detected.

Supplementary Figure 4. Percentage of classical, intermediate and non-classical monocytes within total monocyte population in the Microx (A) and Immunofatigue (B) cohort. Bars represent the mean and error bars indicate standard deviations, no significant differences were detected.

Supplementary Figure  5. Analysis of total T-cells, CD3+CD4+, CD3+CD8+, B-cells and NK cells in PBMCs of the Microx (A, top panel) and Immunofatigue (B, bottom panel) cohorts. Bars represent the mean and error bars indicate standard deviations. Except for CD3+CD8+ in Microx  p < 0.05  (*), no significant differences were detected.

OLE objects.

OLE object

Supplementary Figure 6. A Pearson correlation for G3-YSD and 3p-RNA induced IFN-I bioactivity for the Microx (A, r = -0.10, p = 0.56) and Immunofatigue cohort (B, r = 0.28, p = 0.19)
